# Evaluation of the Physicochemical and Functional Properties of Aquasoya (*Glycine max* Merr.) Powder for Vegan Muffin Preparation

**DOI:** 10.3390/foods11040591

**Published:** 2022-02-18

**Authors:** Yoon-Ha Kim, Weon-Sun Shin

**Affiliations:** Department of Food and Nutrition, College of Human Ecology, Hanyang University, 17 Haengdang-dong, Seongdong-gu, Seoul 04763, Korea; yungha2@naver.com

**Keywords:** aquasoya, yellow soybean, egg white replacement, functional properties, vegan muffins

## Abstract

Recent concerns on health and sustainability have prompted the use of legumes as a source of plant-based proteins, resulting in the application of their cooking water as a substitute for egg whites. In this study, the cooking water of yellow soybeans was powdered, and, subsequently, the nutritional and functional characteristics of powders from yellow soybeans (YSP), chickpeas (CHP), and egg whites (EWP) were compared. The main components of these powders (total polyphenol, total carbohydrate, and protein), along with their hydration properties (hygroscopicity, water solubility index, and water/oil holding capacities), and emulsifying and foaming properties, were identified. The muffins prepared with YSP, CHP, and EWP were analyzed to determine their basic characteristics, such as volume, baking loss, and sensory attributes. The results of the powder analyses indicated that YSP was significantly superior to CHP and EWP, particularly in terms of holding capacities, and emulsion and foam stabilities. The sensory evaluation results showed that there was no statistically significant difference in overall acceptance among the muffin samples. Therefore, YSP can be used as an alternative to CHP or EWP, and applied as a novel ingredient in various vegan products.

## 1. Introduction

Eggs are one of the most commonly used food ingredients worldwide [1]. They are nutritionally-complete ingredients from an animal origin, and are used in various bakery products due to their remarkable functional characteristics. However, recently, interest in plant-based proteins as an alternative to animal proteins has increased because of the development of vegetarian market [2]. The plant-based protein market could make up to 7.7% of the global protein market by 2030, with a value of over 162 billion dollars, up from 29.4 billion dollars in 2020, according to a new report by Bloomberg Intelligence (2021).

Soybeans are suitable for further development in the food industry because of their sustainable utilization, high nutritional value, relatively low allergy risk, high yield, and low cost [3]. Thus, soybeans are considered a suitable plant-based protein source as an alternative to animal proteins, such as eggs. Chickpeas are mainly composed of resistant starch and water-soluble fibers, and are rich in polyphenols, carbohydrates, and proteins, although the nutritional and structural characteristics vary depending on the variety of chickpea [4]. The cooking water (CW) from legume seeds such as chickpeas (i.e., aquafaba) has been widely used to substitute egg whites because it has excellent functional properties that can create stable foam and emulsion, similar to those of egg whites [5]. Therefore, many previous studies have dealt with the possibility of using CW from chickpeas instead of egg whites. Alajaji and El-Adawy proved that the carbohydrate and protein contents of chickpeas could be used to create stable foam [4]. However, there have been several studies indicating that yellow soybeans are higher in crude protein and fiber contents (40.59 and 7.27 g/100 g, respectively) than chickpeas (20.9 and 4.32 g/100 g, respectively), which are closely related to foamability [2]. In addition, according to the Food and Agriculture Organization (FAO), the world production of soybeans was about 348.7 million tons in 2018, whereas that of chickpeas was about 17.18 million tons. These soybeans are widely used in the manufacture of various foods, and most of their CW is discarded, resulting in enormous disposal costs and extreme water pollution [3]. Thus, several solutions were sought to overcome these problems in a previous study reported by Shim et al. [2]. According to other studies, the nutritional and functional characteristics of CW from yellow soybeans are superior to those from chickpeas, but inferior to those from black soybeans and small black beans. However, since they analyzed the liquid form of CW, it was expected that the characteristics would be different from those of the concentrated and dried CW used in this study [2,3].

Although there are these findings on the usability of soybean CW, the possibility of using CW powder of yellow soybeans has not yet been explored. Therefore, this study utilized the powder of yellow soybeans to determine whether it could replace that of chickpeas and egg whites. In the present study, CW of legumes was powdered to facilitate distribution, and ensure efficient storage [6]. This study contrasts significantly with other studies in that the powder form, which can be commercialized and stored for a long time, was used instead of the liquid form. Yellow soybean powder (YSP), chickpea powder (CHP), and egg white powder (EWP) were prepared for further analysis, and for baking muffins. CHP and EWP were used as controls for comparison with YSP. There have been many studies using CW of chickpeas in manufacturing muffins because the muffins could be used as appropriate food matrices to test the emulsifying and foaming abilities, which are the main characteristics of CW [7]. However, there have been no studies comparing muffins made of powders from different CW. Therefore, this study aims to develop vegan muffins using these powders instead of egg whites by assessing their physicochemical and functional properties and sensory attributes.

## 2. Materials and Methods

### 2.1. Reagents and Materials

Yellow soybeans (variety name of Daewon), wheat flour, soybean oil, canola oil, stevia, baking powder (BP), baking soda (BS), and vanilla extract were purchased from a local supermarket (Seoul, Korea). Canned chickpeas were supplied by F. DIVELLA SPA (Bari, Italy). The brine additive present in the chickpea CW is purified salt. EWP was obtained from EUNSAN FOOD Co., Ltd. (Yeoju, Korea). Total carbohydrate and protein assay kits were obtained from Sigma-Aldrich (St. Louis, MO, USA). All chemicals used were of reagent grade.

### 2.2. Preparation of Powder from Legume CW

#### 2.2.1. Cooking

The CW of legumes was prepared according to the method suggested by Echeverria et al., with slight modifications [3]. Yellow soybeans were rinsed and cooked with tap water at a ratio of 1:4 (*w/v*) for 120 min under low pressure (45 kPa) in an Instant Pot Duo 60 (Double Insight Inc., Ottawa, ON, Canada). After boiling, the CW was collected and cooled to 25 °C. The CW of chickpeas, excluding solid legumes, was also collected from canned chickpeas. The samples were stored at 4 °C until further use.

#### 2.2.2. Spray-Drying

The collected CWs of the samples were dried using an SD-05 pilot-scale spray-dryer (Yoojin Tech Co., Ltd., Yeoju, Korea) equipped with two liquid spray nozzles. The spray-drying conditions were established by the method reported by Caliskan and Nur Dirim, with slight modifications [8]. The temperatures of the inlet and outlet were 180 °C and 95–105 °C, respectively, and the flow rate was 1 kg/h. The obtained powders were stored at 4 °C until use.

### 2.3. Compositional Analysis of Powder from Legume CW

#### 2.3.1. Determination of Total Polyphenol Content

Total polyphenol content (TPC) of the powder was measured using a colorimetric assay with slight modifications [9]. First, 0.1 g of the powder was dispersed in 200 mL of distilled water. Subsequently, a 0.2 N Folin–Ciocalteu reagent (2.5 mL) was mixed with the sample (0.5 mL), followed by a 7.5% sodium carbonate solution (2 mL). The mixture was incubated in the dark at 25 °C for 2 h. The absorbance of the mixture was measured at 760 nm after incubation, using a Synergy HT Multi-Detection microplate reader (BioTek Instruments, Winooski, VT, USA). Gallic acid was used as a standard, and TPC was expressed in g per 100 g of powder.

#### 2.3.2. Determination of Total Carbohydrate Content

Total carbohydrate content (TCC) was determined by a total carbohydrate assay kit (phenol-sulfuric acid method, Takara Bio, CA, USA), following the method suggested by Echeverria et al. [3]. Briefly, 0.1 g of the sample was dissolved in 200 mL of distilled water, and centrifuged at 130,000× *g* for 5 min. Thereafter, the supernatant (0.5 mL) was mixed with a 95% sulfuric acid solution (5 mL). The mixture was placed in a dry oven at 90 °C for 15 min. Subsequently, 5% phenol (1 mL) was added, and the absorbance was measured at 490 nm. Glucose was used as a standard, and TCC was expressed in g per 100 g of powder.

#### 2.3.3. Determination of Protein Content

Protein concentrations were quantified using a bicinchoninic acid protein assay kit (BCA, Thermo Fisher Scientific, Waltham, MA, USA) [10]. Subsequently, 0.1 g of each powder was diluted, 15 times for YSP and CHP, and 50 times for EWP, to maintain the protein concentrations within a specified range. Further, 25 µL of the sample was placed in a 96-well plate. Next, 200 µL of a BCA working reagent was added. After shaking for 30 s, the sample was incubated in a powersonic 505 water bath (Hwashin Technology, Seoul, Korea) at 37 °C for 30 min, and cooled to 25 °C. Finally, the absorbance was measured at 562 nm. Bovine serum albumin (BSA) was used as a reference protein to construct a standard curve. Protein content was also expressed in g per 100 g of powder.

### 2.4. Functional Properties of Powder from Legume CW

#### 2.4.1. Hygroscopicity

Hygroscopicity was evaluated according to the method reported by Jimenez-Sánchez et al. [11]. The sample powder was deposited in a desiccator filled with distilled water (300 mL) at 21 °C and a relative humidity of 69%. The changes in the weight of the powder were recorded every hour for the first 6 h, and every 2 h from 24 to 36 h. The mass of absorbed water is the weight of the powder increased or decreased from 0.5 g over time. All samples were analyzed in triplicate, and the hygroscopicity was calculated using the following equation:

$$\mathrm{Hygroscopicity}\;\left(g/100\;g\right)=\frac{\mathrm{Mass}\;\mathrm{of}\;\mathrm{absorbed}\;\mathrm{water}}{\mathrm{Mass}\;\mathrm{of}\;\mathrm{powder}}\times 100$$

#### 2.4.2. Water Solubility Index

Water solubility index (WSI) was calculated as the percentage of dry solids extracted by evaporating the supernatant of the solution [12]. The sample powder (0.5 g) was dispersed in distilled water (30 mL), and centrifuged in a Combi R-514 centrifuge at 4000 rpm for 30 min (Hanil Scientific Inc., Incheon, Korea). Next, the supernatant (5 mL) was transferred to a pre-weighed beaker, and oven-dried at 105 °C for 3 h. The WSI calculation was performed using the equation:

$$\mathrm{WSI}\;\left(\%\right)=\frac{\mathrm{Mass}\;\mathrm{of}\;\mathrm{powder}\;\mathrm{in}\;\mathrm{supernatant}\;\left(g\right)}{\mathrm{Mass}\;\mathrm{of}\;\mathrm{powder}\;\mathrm{in}\;\mathrm{solution}\;\left(g\right)}\times 100$$

#### 2.4.3. Water- and Oil-Holding Capacities

The water-holding capacity (WHC) and oil-holding capacity (OHC) of the powders were evaluated according to the method reported by Zhang, Xu, and Li, with slight modifications [13]. First, the powder (0.5 g) was dissolved in a pre-weighed centrifugation tube with distilled water (7 mL). After heating at 60 °C for 30 min in the water bath, the mixture was centrifuged at 4000 rpm for 30 min. Subsequently, the supernatant was removed, and the mass of the centrifugation tube containing the wet samples was weighed. The calculation of WHC values was conducted using the equation:

$$\mathrm{WHC}\;(g/g)=\frac{M_{2}-\left(M_{1}+M\right)}{M_{1}}$$

where M, $M_{1}$, and $M_{2}$ correspond to the weights of the centrifugation tube, powder, and wet powder, respectively.

The OHC was determined as follows. The powder (1.0 g) and canola oil (10 mL) were mixed in a centrifugation tube for 30 s using a Vortex-Genie 2 mixer (Scientific Industries Inc., Portland, OR, USA). The sample was then centrifuged at 2500× *g* for 25 min. The resulting supernatant oil was transferred to a graduated cylinder, and the volume was measured. The calculation of OHC values was performed using the equation:

$$\mathrm{OHC}\;(\mathrm{mL}/g)=\frac{\left(V_{1}-V_{2}\right)}{M}$$

where M corresponds to the weight of the powder, whereas $V_{1}$ and $V_{2}$ are the volumes of the initial oil and free oil after centrifugation, respectively.

#### 2.4.4. Emulsifying Properties

The emulsion capacity (EC) and emulsion stability (ES) were evaluated following the method provided by Lafarga, Villaró, Bobo, and Aguiló-Aguayo [14]. Briefly, the powder (0.6 g) was dispersed in distilled water (8 mL) to obtain a final protein concentration of 3%, and adjusted to pH 5.0 with HCl (0.5 N), which is similar to that of muffins. Then, canola oil (12 mL) was added, and the solution was mixed for 30 s. The emulsion was homogenized at 14,000 rpm for 2 min using a T-25 digital ULTRA-TURRAX homogenizer (IKA, Saufen, Germany), and centrifuged at 3000× *g* for 15 min. The volume of the emulsion was determined using a graduated falcon. The calculation of EC was conducted using the equation:

$$\mathrm{EC}\;\left(\%\right)=\frac{V^{E}}{V^{T}}\times 100$$

where $V^{E}$ and $V^{T}$ are the volume of emulsion after centrifugation, and the total volume, respectively.

Next, the emulsion was heated to 85 °C for 10 min, and cooled to 25 °C to evaluate ES. After heating, the volume of the emulsion was measured using a graduated falcon. Subsequently, the emulsion was centrifuged at 3000× *g* for 10 min. The calculation of ES was conducted using the equation:

$$\mathrm{ES}\;\left(\%\right)=\frac{V^{H}}{V^{i}}\times 100$$

where $V^{H}$ and $V^{i}$ are the volume of emulsion after heating, and the initial emulsion, respectively.

ES was also evaluated using a Turbiscan MA2000 device (Formulaction, Ramonville-Saint-Agne, France), which consists of a detection head equipped with a near-infrared light source (880 nm). The device was operated by scanning the sample length while recording the backscatter. The light source of the device scanned the sample from top to bottom, while measuring the percentage of light backscattered or transmitted every hour for three days at 25 °C.

The mean droplet size of the samples was measured by dynamic light scattering using an ELSZ-1000 particle diameter analyzer (Otsuka Electronics Co., Ltd., Osaka, Japan) at 25 °C. After diluting the samples 1000 times in distilled water, they were injected directly into the chamber of the device.

#### 2.4.5. Foaming Properties

The foam capacity (FC) and foam stability (FS) were assessed according to the method suggested by Toews and Wang [15]. The powder (0.3 g) was dissolved in distilled water (10 mL) at a final protein concentration of 3%, and adjusted to pH 5.0 with HCl (0.5 N). The solution was mixed for 30 s using the vortex mixer. Thereafter, the mixture was homogenized at 10,000 rpm for 1 min. The generated foam was measured immediately after homogenization. The FC was calculated using the equation:

$$\mathrm{FC}\;\left(\%\right)=\frac{V_{foam,\;\;t=1}-V_{liquid}}{V_{liquid}}\times 100$$

where $V_{foam,\;t=1}$ corresponds to the foam volume at time *t* = 1 min, and $V_{liquid}$ is the volume of the initial liquid.

The generated foam was also measured at 25 °C for 10, 30, 60, 90, and 120 min after homogenization to evaluate the FS, which was calculated using the equation:

$$\mathrm{FS}\;\left(\%\right)=\frac{V_{foam,t}}{V_{foam,t=1}}\times 100$$

where $V_{foam,t}$ is the volume of foam at times *t* = 1, 10, 30, 60, 90, and 120 min.

In addition, the foam structure was analyzed using a Krüss Dynamic Foam Analyzer DFA 100 (Krüss, Germany). The foam was obtained by spraying air in a 40-mm tempered glass column with a filter (12–25 µm) at a set gas flow rate of 0.3 L/min for 15 s [16]. The foam structure was indicated by the bubble size and count. The images of foam structure were captured at 15, 180, and 900 s during the foam creation process.

### 2.5. Preparation of Muffins

Muffins are one of the representative bakery products made from eggs. Muffins were selected as a food matrix to evaluate the emulsifying and foaming properties of the CW powders. The muffins were prepared by following the recipe provided by Rahmati and Tehrani [17], with slight modifications. The ingredients required are 70 g wheat flour, 4 g powder (each for YSP, CHP, and EWP), 25 g soybean oil, 15 g stevia, 35 g water, 1 g BP, 0.5 g BS, and a small amount of vanilla extract. First, each aquasoya powder was dissolved in water, and whipped with stevia for 5 min using a household mixer (MotorMillions Electric Industries Co., Ltd., Dongguan, China). Subsequently, the sieved wheat flour, BP, BS, soybean oil, and vanilla extract were added. Finally, 70 ± 2 g of the batter was placed in a muffin mold and baked at 175 °C for 25 min in an electric oven (Daeyung Bakery Machinery Ind. Co., Ltd., Seoul, Korea). The baked muffins (final protein concentration of approximately 7.0%) were cooled to ambient temperature (25 °C), and stored at −18 °C until further use.

### 2.6. Determination of Muffin Characteristics

#### 2.6.1. Volume, Baking Loss, and Moisture

The characteristics of muffins, such as volume, baking loss, and moisture content, were assessed using the methods reported by Rahmati and Tehrani [17]. The volume was measured using the sesame seed displacement method, and expressed as a specific volume. The specific volume was calculated using the equation:

$$\mathrm{Specific}\;\mathrm{volume}\;\left(\mathrm{mL}/g\right)=\frac{\mathrm{Volume}\;\mathrm{of}\;\mathrm{muffin}\;\left(\mathrm{mL}\right)}{\mathrm{Weight}\;\mathrm{of}\;\mathrm{muffin}\;\left(g\right)}$$

The baking loss was measured by weighing the muffins 1 h after baking, and was calculated using the equation:

$$\mathrm{Baking}\;\mathrm{loss}\;\left(\%\right)=\frac{\left(B-C\right)}{IW}\times 100$$

where *B* and *C* are the weights of the batter before and after baking, respectively, and *IW* is the initial moisture content of the batter.

The moisture content was measured by drying the muffins in an air oven at 105 °C, according to the air oven gravimetric method AACC 44-15A.26 (AACC International, Eagan, MN, USA).

#### 2.6.2. Color

The color of the muffins (crumb and crust) was determined by measuring the parameters (L*, a*, and b*) using a CR-400 Chroma Meter (Konica Minolta, Tokyo, Japan). The device was calibrated before each analysis using a white standard ceramic tile (Reference No. 1353123, Y = 92.7, x = 0.3133, and y = 0.3193). The parameter L* represents lightness in the range of 0–100 from black to white, a* represents redness on a −a* to +a* scale from green to red, and b* represents yellowness on a −b* to +b* scale from blue to yellow. The total color differences (ΔE*) between the control muffins (with CHP and EWP) and the test muffins (with YSP) were calculated as follows [18]:

$${\Delta E}^{*}=\sqrt{{\left({\Delta L}^{*}\right)}^{2}+{\left({\Delta a}^{*}\right)}^{2}+{\left({\Delta b}^{*}\right)}^{2}}$$

Using the color parameters, the differences between the first measurement and the time-dependent measurement (1 month later) were calculated as the color difference values ΔL*, Δa*, and Δb* [19]. The values were interpreted as follows: ΔE* < 1, color differences were not evident to human eyes, 1 < ΔE* < 3, color differences were not recognized by human eyes, and ΔE* > 3, color differences were evident to human eyes [18].

#### 2.6.3. Texture

The textural characteristics (hardness, adhesiveness, cohesiveness, springiness, gumminess, and chewiness) were measured by the AACC Approved Method (74–09), using a TMS-Pilot Food Texture Analyzer (Food Technology Corporation, Sterling, VA, USA). The textural parameters were calculated by the TPA program. Hardness 1 and 2 are defined as the maximum forces in the first and second press sections, respectively. Adhesiveness means the negative force area for the first bite, and cohesiveness is how well the food withstands a second deformation relative to its resistance under the first deformation. Also, springiness is the height at which food can recover between the end of the first bite and the beginning of the second bite. Gumminess is defined as hardness × cohesiveness, and chewiness is defined as hardness × cohesiveness × springiness [20]. The compression force reading was taken at the point on the curve where the sample had been compressed by 25%. The muffin samples were prepared by cutting the crumb center into cube-shaped blocks of dimensions 2.0 cm × 2.0 cm × 2.0 cm. The test speed was 60 mm/min with a 25% deformation of the original cube height, and a trigger force of 0.15 N was selected.

#### 2.6.4. Surface Area and Pore Size

The Brunauer–Emmett–Teller (BET) surface area and pore size distribution were determined using a 3 Flex 3500 surface area and pore size analyzer (Micromeritics Instrument Corporation, Norcross, GA, USA), following the method reported by Zhang et al., with slight modifications [21]. The device is a high-performance adsorption analyzer for measuring the surface area, pore size, and pore volume of the powder. It is also ideal for gas or vapor adsorption analysis of microporous (<2 nm) and mesoporous (2–50 nm) substances. The isothermal data collection started in the 10^−6^ Torr range (10^−9^ relative pressure range for nitrogen). Each muffin sample was dried before analysis using a FD 850 freeze-dryer (IlShin BioBase, Jungnang-gu, Seoul, Korea) at −80 °C. Subsequently, the sample was crushed into small pieces, and filtered through a 300−µm−aperture.

#### 2.6.5. Sensory Evaluation

Sensory analysis of the muffins was conducted by 20 trained panelists. The panels, who had been trained for the evaluation of vegan products, participated after seeing the recruitment announcement. A nine-point hedonic scale from 1 (dislike extremely) to 9 (like extremely) was employed to evaluate all sensory attributes, including appearance (color, air cell uniformity, and loaf volume), flavor (beany flavor and after-flavor), texture (moistness and stickiness), and overall acceptability. The meaning of each descriptor was explained to all panelists. Each muffin sample (15 g) was randomly arranged in a three-digit number. The panelists tested the samples and rinsed their mouths thoroughly with water before testing the next sample. This study was approved by the Hanyang University Institutional Review Board (IRB number HYUIRB-202108-003).

### 2.7. Statistical Analysis

The results are presented as the mean ± standard deviation (SD) of the triplicate tests. Statistical analysis was conducted using SPSS (version 26.0; SPSS Inc., Chicago, IL, USA). Significant differences were identified by one-way analysis of variance (ANOVA), followed by Duncan’s post-hoc test (*p* < 0.05).

## 3. Results and Discussion

### 3.1. Compositional Analysis

#### 3.1.1. Total Polyphenol Content

Natural phenols have certain health benefits, such as the elimination of free radicals, inhibition of oxidases, and activation of antioxidants [22]. As shown in Table 1, EWP had the highest level of TPC (2.14 g/100 g), followed by YSP (1.23 g/100 g) and CHP (0.85 g/100 g). Similarly, Xu, Yuan, and Chang reported that all three species of yellow soybeans (for example, Proto, Korada, and Tofuyi) had significantly higher TPC values than chickpeas (*p* < 0.05) [23]. Polyphenols in dry legumes are transferred into the CW during thermal processes because phenolic contents mainly exist in the legume coat [3]. Yellow soybeans are generally smaller than chickpeas, and have thinner shells. Hence, they have a large contact area with water, and internal nutrients can be eluted easily [3]. Furthermore, most phenolic compounds are non-conjugated, resulting in the decomposition of polyphenols during heat treatment [24]. Egg whites are one of the food ingredients with very low polyphenol content, but in this study, the lower TPC of YSP and CHP than EWP might be derived from thermal treatment, such as cooking and spray-drying [25]. According to Bednarska and Janiszewska-Turak (2020), the drying temperature and the polyphenol content are proportional [26]. Although the actual drying temperature of EWP used in this study was not accurate, it was expected that the inlet/outlet temperatures of EWP might be higher than those of YSP and CHP during spray-drying. In addition, the CW powder contained more than 10 times higher nutritional components than the liquid CW, and had superior or similar functional properties. The previous study on liquid CW from pulses reported that the TPC of yellow soybeans and chickpeas were 0.07 and 0.03 g/100 g, the TCC were 4.74 and 3.28 g/100 g, and the protein contents were 1.51 and 0.39 g/100 g [3]. This difference would be due the concentration and drying process. Therefore, the validity of using the CW powder instead of the liquid CW could be confirmed.

#### 3.1.2. Total Carbohydrate Content

As expected, Table 1 shows that EWP had the lowest TCC value (2.75 g/100 g) among the powders, similar to the results of Pérez-Reyes, Tang, Barbosa-Cánovas, and Zhu [27]. In contrast, YSP exhibited the highest TCC value (52.37 g/100 g), followed by the CHP (46.91 g/100 g), indicating a statistically significant difference between the legumes (*p* < 0.05). According to Stantiall, Dale, Calizo, and Serventi [28], the carbohydrates in CW of legumes are water-soluble, including high-molecular-weight (such as soluble fiber) and low-molecular-weight (such as sucrose, raffinose, or stachyose) ones. They were leached into the CW, and concentrated during spray-drying, resulting in a high TCC level. This result is in good agreement with that of National Institute of Agricultural Sciences of Korea (2016), in that chickpeas had lower water-soluble carbohydrate content than yellow soybeans. Therefore, the contents of water-soluble carbohydrates in legumes affected the TCC of the final powder.

#### 3.1.3. Protein Content

Proteins present in legumes in large quantities are associated with functional properties, such as emulsifying and foaming abilities. EWP had higher protein content (81.91 g/100 g) than YSP and CHP (23.11 g/100 g and 23.07 g/100 g, respectively) (Table 1). However, these results were very high compared to the protein data of dried yellow soybeans (16.21 g/100 g) and chickpeas (17.27 g/100 g), obtained from the Korean Food Composition Table, 9th revision (Wanju, Korea, 2016). The reason for these high values is that the powders were concentrated through the spray-drying process. The number of proteins found in the CW was correlated with the loss of proteins from legumes during cooking [28]. According to research by Alsalman, Tulbek, Nickerson, and Ramaswamy [29], protein content in CW is positively correlated with cooking time. Therefore, the longer cooking time (120 min) of yellow soybeans than the previous one (20–90 min) increased the protein content of yellow soybean CW [4,5,24]. Several studies have shown that globulin, the most abundant protein in legumes, has better WHC and OHC than ovalbumin, the most abundant protein in egg whites [30]. According to the data (not provided), the isoelectric points (pI) of the powder (YSP, CHP, and EWP) were pH 5.0–5.2. Since proteins are generally least soluble at pI, the lack of electrostatic repulsion and protein aggregation would occur if the pH is about 5.0. The protein solubility can represent the function and activity of biocatalysts which are mainly composed of recombinant proteins [31]. The lowest protein solubility at pI is due to the minimal intermolecular electrostatic forces, and less interaction between protein molecules and water, resulting in increased protein–protein interaction [32].

### 3.2. Functional Properties

#### 3.2.1. Hygroscopicity and Water Solubility Index

**Figure 1:**
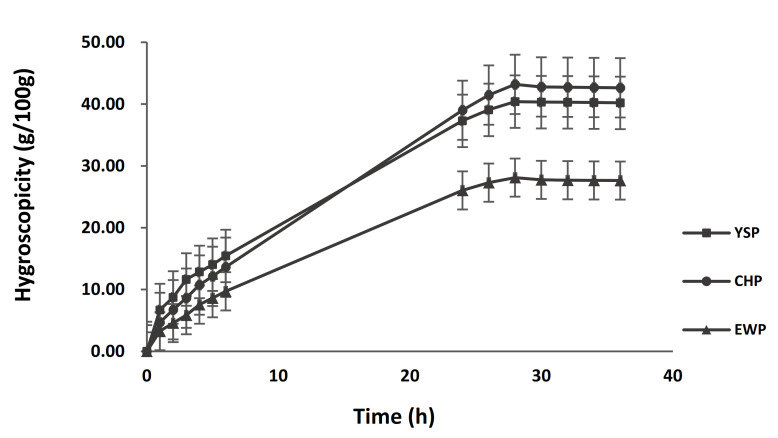
Hygroscopicity of yellow soybean powder (YSP), chickpea powder (CHP), and egg white powder (EWP) measured for 36 h. All values are expressed as mean ± standard deviation (*n* = 3).

Hygroscopicity affects the fluidity and water absorption capacity of the powder, and influences the shelf life of the products [33]. Figure 1 illustrates an increase in water absorption of each powder within 36 h. All of the samples exhibited a steady increase in water absorption until 28 h, and a slight decrease before reaching equilibrium (30–36 h). The decrease in water absorption was attributed to the lack of space within the powder matrix combined with water [34]. According to Table 1, CHP showed the highest hygroscopicity (38.80 g/100 g), followed by YSP (35.34 g/100 g) and EWP (25.88 g/100 g). The significantly higher values for CHP and YSP than those for EWP could be related to TCC. Carbohydrates enable the binding of hydrogen groups with hydrogen atoms in water molecules, thereby improving the hygroscopicity value [35]. Additionally, CHP was highly hygroscopic because of the branched structure, such as in amylopectin, which may promote hydrogen bonding [36]. Huang et al. also demonstrated that chickpeas contain higher amylopectin than yellow soybeans [37]. Thus, CHP had the highest hygroscopicity throughout the process.

WSI is often used to evaluate powder reconstitution. According to the data presented in Table 1, EWP showed a significantly higher value (94.75%) than the other powders, similar to that observed in a previous study by Li, Sheng, and Jin [38]. However, there was no significant difference (*p* > 0.05) between the WSI values of YSP (81.06%) and CHP (80.28%). The higher solubility of EWP could be related to the connection between phenolic compounds and proteins, allowing polyphenols to bond between two protein molecules, and reducing protein aggregations [5]. Furthermore, the reason YSP and CHP had lower WSI than EWP is that the conformational changes of proteins occurred by the extended thermal treatment (cooking yellow soybeans for 120 min, and chickpeas for 30–60 min), which promotes protein denaturation, and induces the formation of protein aggregates, which limit the interaction between proteins and water [39].

#### 3.2.2. Water- and Oil-Holding Capacities

The WHC and OHC are important parameters for texture development, as listed in Table 1. The WHC of YSP was significantly higher (1.45 g/g) than that of CHP (1.00 g/g) and EWP (0.95 g/g). The WHC reflects the capability of proteins and carbohydrates to prevent water from being released or expelled from their three-dimensional structures [40]. Therefore, it is directly proportional to the protein content and denaturation temperature. According to Traynham et al. (2007), the legume proteins can be more easily combined with water molecules than egg white proteins [41]. Thus, the denaturation temperature of legume proteins is generally higher than that of egg white proteins. This result is in good agreement with previous studies reported by Ghribi et al. (2015) and Ahmed et al. (2007), indicating that the denaturation temperatures of legume proteins and egg white proteins are 83.8 °C and 74.1 °C [42,43]. Previous studies have also revealed that water-soluble carbohydrates contribute to high water-binding capacity [44]. Thus, the relatively low TCC of EWP might have led to low WHC.

The OHC also had a similarity to that of WHC. YSP had slightly higher OHC (2.31 mL/g) than CHP (2.23 mL/g) and EWP (2.13 mL/g). A previous study demonstrated that the high TCC value can be related to an increase in OHC [5]. Both water soluble and insoluble polysaccharides are able to absorb oil. The significant difference between YSP and CHP is due to cooking time. In this study, the yellow soybeans were cooked for 120 min longer than the canned chickpeas (30–60 min). The previous study by Alsalman, Tulbek, Nickerson, and Ramaswamy revealed that the OHC value is directly proportional to the cooking time [29]. Furthermore, longer cooking times could lead to protein denaturation, thereby increasing the OHC values due to exposure of the hydrophobic regions [29].

#### 3.2.3. Emulsifying Properties

Proteins are well-known to be associated with emulsifying properties because they can quickly adsorb to the interface, and then lower the interfacial tension of the emulsion system. Proteins usually vary in their ability to form stable emulsions, depending on their type [45]. Table 1 lists EC and ES values of the powder samples. Although the protein content of EWP was four times higher than that of YSP and CHP (Table 1), there was no significant difference in EC (*p* > 0.05). According to Giovannelli et al. (2021), it was estimated that carrier agents were used during the spray-drying process of EWP [46]. As the concentration of carriers increased, it was expected that the depletion flocculation occurred, and the emulsifying properties decreased [47]. In addition, the high solubility of EWP had no impact on the emulsifying capacity. These results are consistent with those reported by Wong and Kitts [48]. They revealed that the increasing of protein solubility may not improve the EC, since excessive protein content cannot migrate to the o/w interface.

The ES value is influenced by several physical interdependent factors, such as cream formation, flocculation or aggregation, and coalescence [49]. Emulsions made with EWP (81.32%) and YSP (77.87%) were more stable than those made with CHP (69.86%). Nesterenko et al. revealed that a large particle size reduces ES [50]. However, in this study, the YSP emulsion had a larger particle size and better stability than the CHP emulsion. This is closely related to the TCC values (Table 1), particularly the polysaccharide content. The excessively increased concentration of non-adsorbed polysaccharides may increase the instability due to their depletion flocculation mechanisms [51]. In addition, the PDI (polydispersity index) value of the EWP-stabilized emulsion was the smallest, followed by YSP and CHP (data not shown). According to Zhang et al. (2015), the smaller PDI can be related to the higher ES, because the PDI value represents the particle size distribution of the emulsion droplets [52]. Consequently, the statistically significant (*p* < 0.05) ES values were validated based on these results.

**Figure 2:**
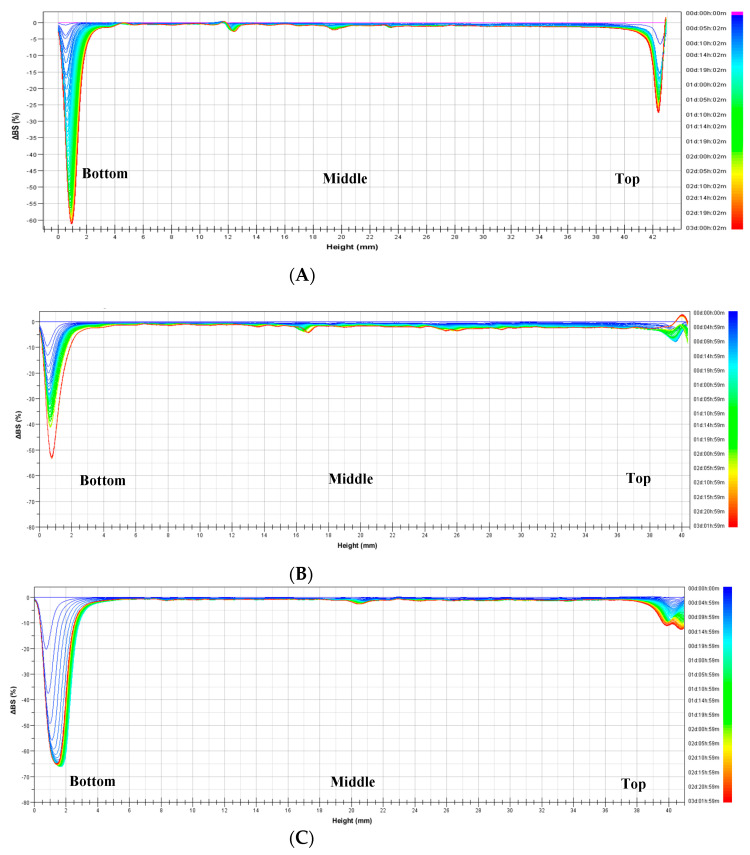
Delta backscattering (ΔBS) of the emulsion from (**A**) yellow soybean powder (YSP), (**B**) chickpea powder (CHP), and (**C**) egg white powder (EWP).

The delta back scattering (ΔBS) data obtained by Turbiscan indicate the ES of each height part (top, middle, and bottom) of the emulsion (Figure 2). The ΔBS means a function of the height of the measurement cell (axis of abscissas) for different times in hours. Over time, the ΔBS values decreased because the particles moved to the top, resulting in uneven aggregates of more than 0.6 µm in the middle. In addition, the ΔBS values decreased because of the creaming caused by the strong cohesion at the top. In the middle, CHP showed more aggregation than YSP, which reduced the ΔBS values of YSP. However, YSP exhibited more creaming than CHP at the top. These results are attributed to the larger droplet size of the emulsion with YSP than that with CHP. Therefore, the YSP emulsion could be used to manufacture muffins with a fluffy texture because it showed more creaming than CHP and EWP.

#### 3.2.4. Foaming Properties

**Figure 3:**
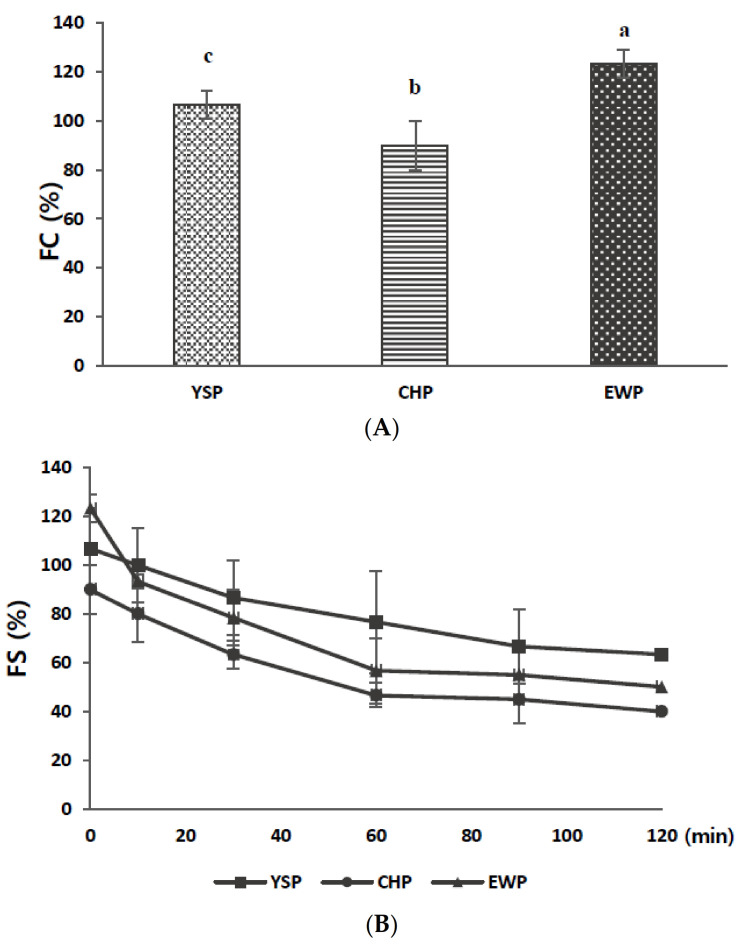
(**A**) Foam capacity (FC) and (**B**) foam stability (FS) of yellow soybean powder (YSP), chickpea powder (CHP), and egg white powder (EWP). All values are expressed as mean ± standard deviation (*n* = 3). Means with different superscripts (a–c) in each column are significantly different (*p* < 0.05) according to Duncan’s test.

The FC and FS values of the powders are illustrated in Figure 3A,B, respectively. First, EWP exhibited the highest FC value (123.33%), followed by YSP (106.67%) and CHP (90.00%), similar to the result reported by Stantiall, Dale, Calizo, and Serventi (2018) (Figure 3A). Because FC is affected by protein concentration and solubility, the obtained results are consistent with those of protein content and WSI (Table 1) in this study. In the process of foam formation, proteins dissolved in liquid quickly move to the interface between the dispersed gas and liquid as air enters, thereby adsorbing and changing the structure on the interface. The protein concentration, protein adsorption intensity, and stability are not necessarily proportional. This is because it is also affected by the protein structure and surface charge. Finally, the lamellae viscosity can be controlled by environmental factors such as sugar and polysaccharides. Therefore, FC is related to pH values because pH acts as a protein solubility control factor [53]. Furthermore, low pH can reduce the protein flexibility and protein solubility by exposing the hydrophobic core of proteins, preventing the protein from diffusing to the air–water interface [54]. Thus, we confer that CHP with the lowest WSI had a lower FC value than YSP and EWP.

In contrast to the FC results, the FS values declined consistently as the foam was created. Foam with YSP was the most stable at all times (from 10 to 120 min) because YSP and CHP, isolated from legumes, had higher carbohydrate contents than EWP [27]. Moreover, TCC can stabilize foams by modifying the viscosity of the aqueous continuous phase, increasing the film thickness, and limiting the movement of bubbles [55].

**Figure 4:**
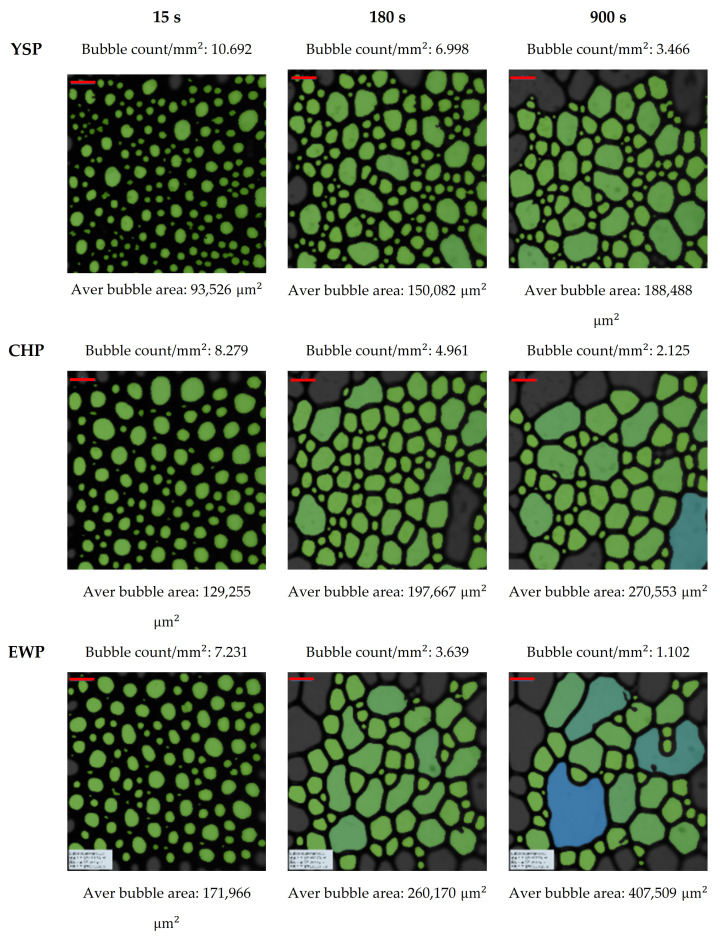
Images of foam structures obtained at 15, 180, and 900 s from yellow soybean powder (YSP), chickpea powder (CHP), and egg white powder (EWP). The red bar at the top of each image means a scale bar, and represents 1 mm.

In addition, the FS can be described by foam structure images. The foam structure was expressed in terms of the bubble size and count, as shown in Figure 4. All the samples showed similar changes over time (from 15 to 900 s), increasing the bubble size, and decreasing the bubble count. When they reached 900 s, it was confirmed that the overall foam became unstable, as bubbles for all samples expanded and burst. The expanded and burst bubbles are expressed in blue (Figure 4).

### 3.3. Characteristics of Muffins

#### 3.3.1. Volume, Baking Loss, and Moisture

The difference in specific volume among the muffin samples was statistically insignificant (*p* > 0.05), as shown in Supplementary Figure S1 and Table 2. As reported by Dhull et al. (2020), the rheological properties of gluten can affect the specific volume of bread. Therefore, in this study, it was speculated that there being no significant difference in the volume of the muffins was because of the same amount of flour used for making the muffins [56]. According to a previous report, FC can also directly affect the loaf volume of baking products [57]. However, in this study, FC was higher in the order of EWP, YSP, and CHP (Figure 3A), but there was no significant difference in the specific volume of muffins. This indicated that FS can be an important factor due to its integrity of interface and lamellar viscosity. The half-life of EWP-stabilized foam was 180 s, but that of both YSP and CHP was 300 s (data not shown). These results suggested that YSP and CHP stabilized the foam, and settled the solid network during baking.

However, there was a significant difference in baking loss values. The muffins with YSP showed lower weight reduction after baking than those with CHP and EWP. The baking loss is closely related to the WHC values because it is affected by moisture absorption and retention [54]. Therefore, the baking loss is low when the WHC is high. Furthermore, the moisture content of muffins is consistent with the WHC results presented in Table 1. Thus, the WHC is directly proportional to the moisture content.

#### 3.3.2. Color

The color parameters of the muffins were significantly different (*p* < 0.05), as listed in Table 3. The colors of the crumb and crust of EWP muffins showed the highest L*, a*, and b* parameters, followed by the CHP and YSP muffins. The differences were observed between the crumb and crust values because the carbohydrate components present on the surface are easily browned by heating [58]. In addition, the color differences (ΔE*) were statistically significant, meaning that the color changes were clearly observed over time. In all the samples, the differences in parameters resulted in ΔE* values much higher than 3, indicating a distinct color difference visible to the human eyes. This could be attributed to the Maillard reaction occurring during baking [59]. Although the baking ingredients were the same, it was considered that the Maillard reaction was further promoted in YSP because of its high TCC.

#### 3.3.3. Texture

The evaluated texture characteristics (hardness, adhesiveness, cohesiveness, springiness, gumminess, and chewiness) were not significantly different among the muffin samples (Table 4). According to a previous study, hardness, gumminess, and chewiness are closely related to the hygroscopicity [56]. This is because the hygroscopic compounds can affect the gelation and retrogradation of starch, thereby enhancing the parameters. However, in this study, the hygroscopicity was high, in order, of CHP, YSP, and EWP (Figure 1), but there was no significant difference in texture characteristics. As mentioned above, this result was estimated due to the rheological properties that acted similarly to all muffins. Furthermore, the EWP muffins exhibited the highest hardness, gumminess, and chewiness values because of the increased protein entanglement in the networks, which led to the reinforcement of the crumb walls surrounding the air cells [60]. In addition, the elastic networks developed by the proteins present in batters directly influenced the cohesiveness and springiness of the muffins [61].

#### 3.3.4. Surface Area and Pore Size

**Figure 5:**
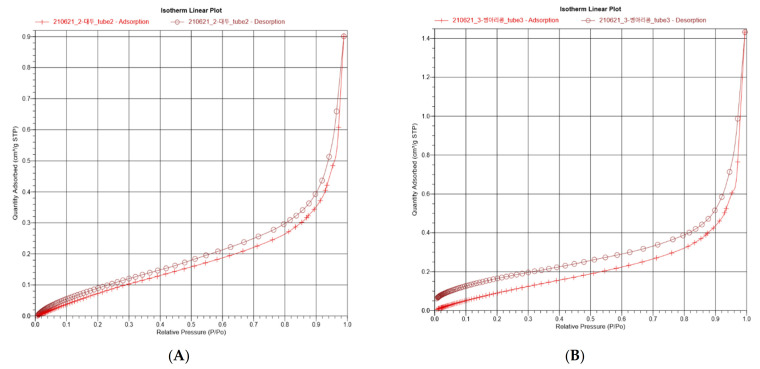

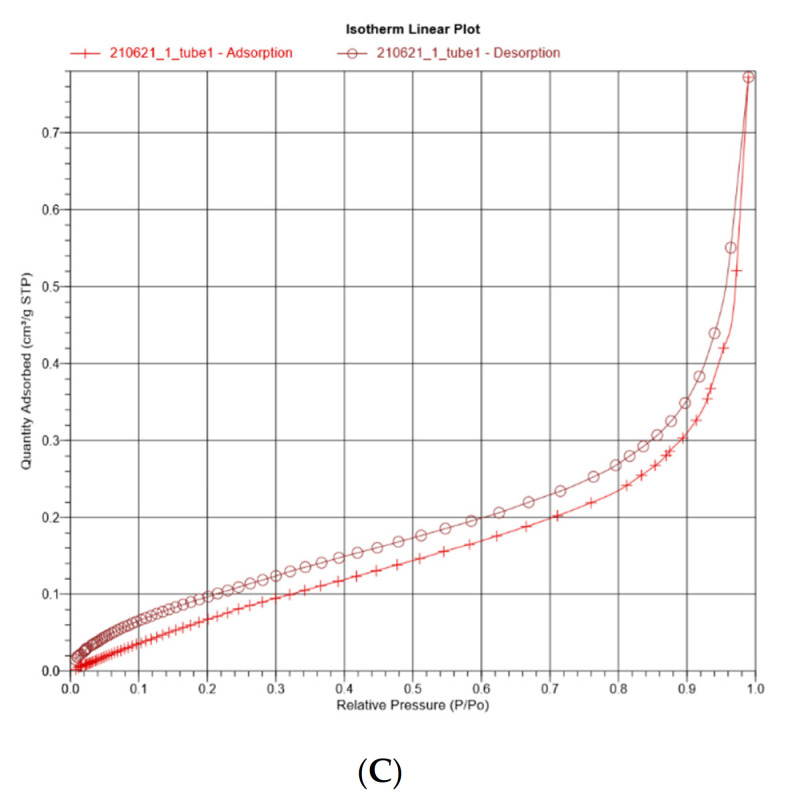
Isotherm linear plots explained by correlation between relative pressure and quantity adsorbed from (**A**) yellow soybean powder (YSP), (**B**) chickpea powder (CHP), and (**C**) egg white powder (EWP).

The $N_{2}$ adsorption–desorption isotherm graphs exhibited similar curves (Figure 5). It was confirmed that the quantity adsorbed increased with increasing relative pressure, as demonstrated by Wang et al. [62]. All the curves showed a steep rise after 0.9 P/Po, irrespective of the legume type. The surface area and adsorption amount are known to be directly proportional to each other. However, the actual adsorption is affected by the pore distribution and pore size of the adsorbents, which was also determined in this study [63]. The pore size distribution curve derived from the adsorption branch using the Barrett–Joyner–Halenda method revealed a uniform pore size of the muffins with YSP (33.946 Å), CHP (35.584 Å), and EWP (33.133 Å). The BET surface area (m²/g) and total pore volume (cm³/g) of the muffins were 0.5863 m²/g and 0.000532 cm³/g for YSP, 0.5815 m²/g and 0.00656 cm³/g for CHP, and 0.5004 m²/g and 0.000469 cm³/g for EWP. Thus, the yellow soybeans can be applied to the bakery products as a substitute for chickpeas and egg whites.

#### 3.3.5. Sensory Evaluation

The sensory analysis results are presented in Table 5. There were no statistically significant differences among the muffins in terms of any of the descriptors. After-flavor can be defined as a degree of taste or aroma after swallowing. Moistness is the amount of moisture perceived when tasting, and stickiness is the degree of stickiness perceived when chewing. The evaluated attributes were found to be closely related to each other. In particular, overall acceptability was greatly influenced by flavor (beany flavor and after-flavor). The acceptability was also strongly correlated with appearance, such as air cell uniformity and loaf volume. These results are in a good agreement with a previous study reported by Fribourg et al. (2020) [64]. Thus, the probability of using YSP in vegetarian products can be higher than that of using CHP and EWP.

## 4. Conclusions

The purpose of this study is to analyze the physicochemical and functional properties of powder from yellow soybean CW, and evaluate the basic characteristics of muffins prepared with this. The yellow soybean powder (YSP) used in our study can be recommended as an effective substitute for CHP and EWP. In fact, the functional properties of YSP, such as emulsification and foamability, were superior to those of EWP and CHP. Furthermore, there was no significant difference when comparing the texture and sensory attributes of muffins made of YSP, CHP, and EWP. Accordingly, it is concluded that YSP can replace CHP and EWP when making the final bakery products without affecting consumer preference. Our study provides a better understanding of the available plant-based proteins as replacements for egg whites, and suggests the need for further research in the future. The next step of this study should be building up the production scale to obtain YSP at the industrial level.

## Figures and Tables

**Table 1:** Total polyphenol content (TPC), total carbohydrate content (TCC), protein, hygroscopicity, water solubility index (WSI), water-holding capacity (WHC), oil-holding capacity (OHC), emulsion capacity (EC), emulsion stability (ES), and mean droplet size of yellow soybean powder (YSP), chickpea powder (CHP), and egg white powder (EWP).

	YSP	CHP	EWP
TPC (g/100 g)	1.23 ± 0.07 ^b^	0.85 ± 0.09 ^c^	2.14 ± 0.03 ^a^
TCC (g/100 g)	52.37 ± 1.27 ^a^	46.91 ± 0.16 ^b^	2.75 ± 0.36 ^c^
Protein (g/100 g)	23.11 ± 0.15 ^b^	23.07 ± 0.09 ^b^	81.91 ± 0.05 ^a^
Hygroscopicity (g/100 g)	35.34 ± 0.67 ^b^	38.80 ± 0.77 ^a^	25.88 ± 0.41 ^c^
WSI (%)	81.06 ± 2.29 ^b^	80.28 ± 5.49 ^b^	94.75 ± 1.70 ^a^
WHC (g/g)	1.45 ± 0.21 ^a^	1.00 ± 0.13 ^b^	0.95 ± 0.10 ^b^
OHC (mL/g)	2.31 ± 0.06 ^a^	2.23 ± 0.10 ^ab^	2.13 ± 0.06 ^b^
EC (%)	69.90 ± 2.11	68.37 ± 1.52	70.32 ± 0.55
ES (%)	77.87 ± 2.01 ^a^	69.86 ± 2.55 ^b^	81.32 ± 0.51 ^a^
Droplet size (µm)	2.93 ± 0.05 ^a^	1.78 ± 0.10 ^b^	1.31 ± 0.07 ^c^

All values are expressed as mean ± standard deviation (*n* = 3). Means with different superscripts (a–c) in each column are significantly different (*p* < 0.05) according to Duncan’s test.

**Table 2:** Characteristics of aquasoya muffins with yellow soybean powder (YSP), chickpea powder (CHP), and egg white powder (EWP): volume, baking loss, and moisture content.

	YSP	CHP	EWP
Volume (mL/g)	1.48 ± 0.89 ^a^	1.43 ± 0.57 ^a^	1.46 ± 0.29 ^a^
Baking loss (%)	25.58 ± 0.17 ^b^	29.88 ± 0.21 ^a^	30.18 ± 0.30 ^a^
Moisture (g)	11.73 ± 0.00 ^a^	10.14 ± 0.10 ^b^	10.11 ± 0.16 ^b^

All values are expressed as mean ± standard deviation (*n* = 3). Means with different superscripts (a,b) in each column are significantly different (*p* < 0.05) according to Duncan’s test.

**Table 3:** Characteristics of aquasoya muffins with yellow soybean powder (YSP), chickpea powder (CHP), and egg white powder (EWP): colors of the crumb and crust.

Color		YSP	CHP	EWP
Crumb	L*	44.14 ± 0.06 ^c^	48.04 ± 0.11 ^b^	49.90 ± 0.03 ^a^
	a*	3.50 ± 0.17 ^c^	5.04 ± 0.22 ^b^	5.85 ± 0.08 ^a^
	b*	11.76 ± 0.06 ^c^	19.32 ± 0.30 ^b^	21.20 ± 0.10 ^a^
	ΔE*	53.80 ± 0.25 ^a^	52.12 ± 0.13 ^b^	51.09 ± 0.04 ^c^
Crust	L*	38.90 ± 0.23 ^c^	37.86 ± 0.03 ^b^	46.49 ± 0.08 ^a^
	a*	7.37 ± 0.16 ^c^	6.39 ± 0.12 ^b^	10.32 ± 0.11 ^a^
	b*	8.04 ± 0.04 ^c^	7.75 ± 0.19 ^b^	14.68 ± 0.04 ^a^
	ΔE*	58.80 ± 0.24 ^b^	59.69 ± 0.14 ^a^	53.01 ± 0.07 ^c^

All values are expressed as mean ± standard deviation (*n* = 3). Means with different superscripts (a–c) in each column are significantly different (*p* < 0.05) according to Duncan’s test.

**Table 4:** Texture of aquasoya muffins formulated with yellow soybean powder (YSP), chickpea powder (CHP), and egg white powder (EWP).

	YSP	CHP	EWP
Hardness 1	13,403.94 ± 1872.28 ^a^	11,685.53 ± 1861.30 ^a^	14,278.76 ± 2869.67 ^a^
Hardness 2	11,716.77 ± 1361.52 ^a^	10,342.05 ± 1629.78 ^a^	12,779.06 ± 2710.65 ^a^
Adhesiveness	0.06 ± 0.01 ^a^	0.07 ± 0.02 ^a^	0.07 ± 0.00 ^a^
Cohesiveness	0.58 ± 0.03 ^a^	0.59 ± 0.02 ^a^	0.61 ± 0.02 ^a^
Springiness	3.86 ± 0.15 ^a^	3.69 ± 0.27 ^a^	3.92 ± 0.19 ^a^
Gumminess	3.08 ± 0.32 ^a^	2.75 ± 0.47 ^a^	3.52 ± 0.77 ^a^
Chewiness	11.86 ± 0.98 ^a^	10.06 ± 0.94 ^a^	13.75 ± 2.81 ^a^

All values are expressed as mean ± standard deviation (*n* = 3). There was no significant difference in mean values with the same superscript (*p* > 0.05).

**Table 5:** Sensory analysis of aquasoya muffins formulated with yellow soybean powder (YSP), chickpea powder (CHP), and egg white powder (EWP).

Descriptor		YSP	CHP	EWP
Appearance	Color	7.25 ± 1.25 ^a^	7.25 ± 1.41 ^a^	7.25 ± 1.37 ^a^
	Air cell uniformity	6.55 ± 1.73 ^a^	7.00 ± 1.21 ^a^	6.00 ± 1.45 ^a^
	Loaf volume	6.45 ± 1.54 ^a^	6.55 ± 1.50 ^a^	6.55 ± 1.43 ^a^
Flavor	Beany	5.70 ± 1.66 ^a^	6.30 ± 1.75 ^a^	5.50 ± 1.77 ^a^
	After-flavor	5.85 ± 1.50 ^a^	6.30 ± 1.38 ^a^	5.90 ± 1.89 ^a^
Texture	Moistness	5.15 ± 1.93 ^a^	5.65 ± 1.84 ^a^	4.95 ± 2.37 ^a^
	Stickiness	5.50 ± 2.28 ^a^	5.50 ± 1.91 ^a^	5.70 ± 1.98 ^a^
Overall Acceptability		6.35 ± 1.45 ^a^	6.40 ± 1.76 ^a^	6.20 ± 1.79 ^a^

All values are expressed as mean ± standard deviation (*n* = 20, 9-point hedonic scale). There was no significant difference in mean values with the same superscript (*p* > 0.05).

## Data Availability

The data presented in this study are available in the article.

## Supplementary Materials

The following supporting information can be downloaded at: https://www.mdpi.com/article/10.3390/foods11040591/s1, Figure S1: Pictures of muffins cut in half, whole muffins (without meringue), and whole muffins (with meringue) made from yellow soybean powder (YSP), chickpea powder (CHP), and egg white powder (EWP).
