# Supplementary material for: Evaluation of the Physicochemical and Functional Properties of Aquasoya (Glycine max Merr.) Powder for Vegan Muffin Preparation
Source: Foods. 2022 Feb 18;11(4):591. doi: 10.3390/foods11040591 (PMC8870977; doi:10.3390/foods11040591)
Supplement: Supplementary file 1 [file foods-11-00591-s001.zip › foods-1457492-supplementary.pdf]

Supplementary Material:

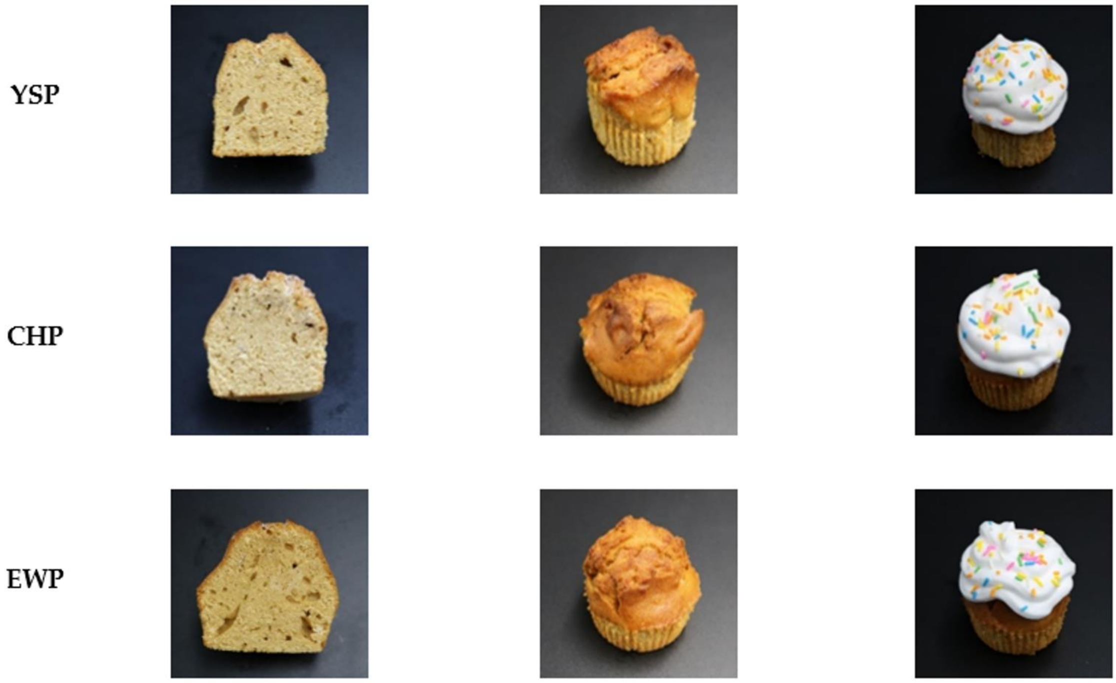

Figure S1. Pictures of muffins cut in half, whole muffins (without meringue), and whole muffins (with meringue) made from yellow soybean powder (YSP), chickpea powder (CHP), and egg white powder (EWP).
